# Supplementary material for: Efficient colonic colonization by Campylobacter jejuni requires the heme receptor ChuA
Source: Infect Immun. 2026 Oct 15;94(11):e00457-26. doi: 10.1128/iai.00457-26 (PMC13750120; doi:10.1128/iai.00457-26)
Supplement: Supplementary file 1 [file iai.00457-26-s0006.pdf]

Figure S1

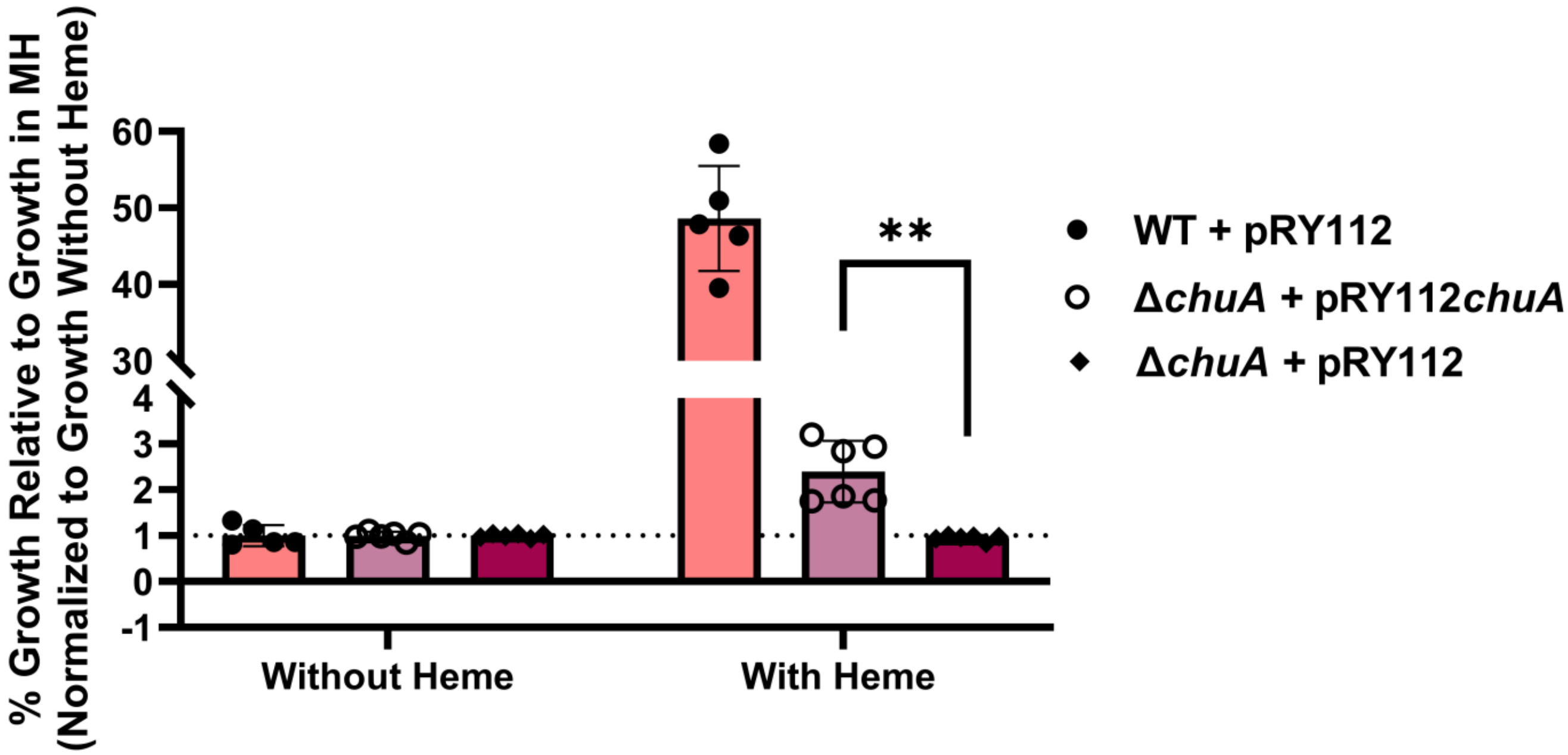

% Growth Relative to Growth in MH
(Normalized to Growth Without Heme)
60
50
40
30
4
3
2
1
0
-1
**
Without Heme
With Heme
WT + pRY112
ΔchuA + pRY112chuA
ΔchuA + pRY112

Figure S2

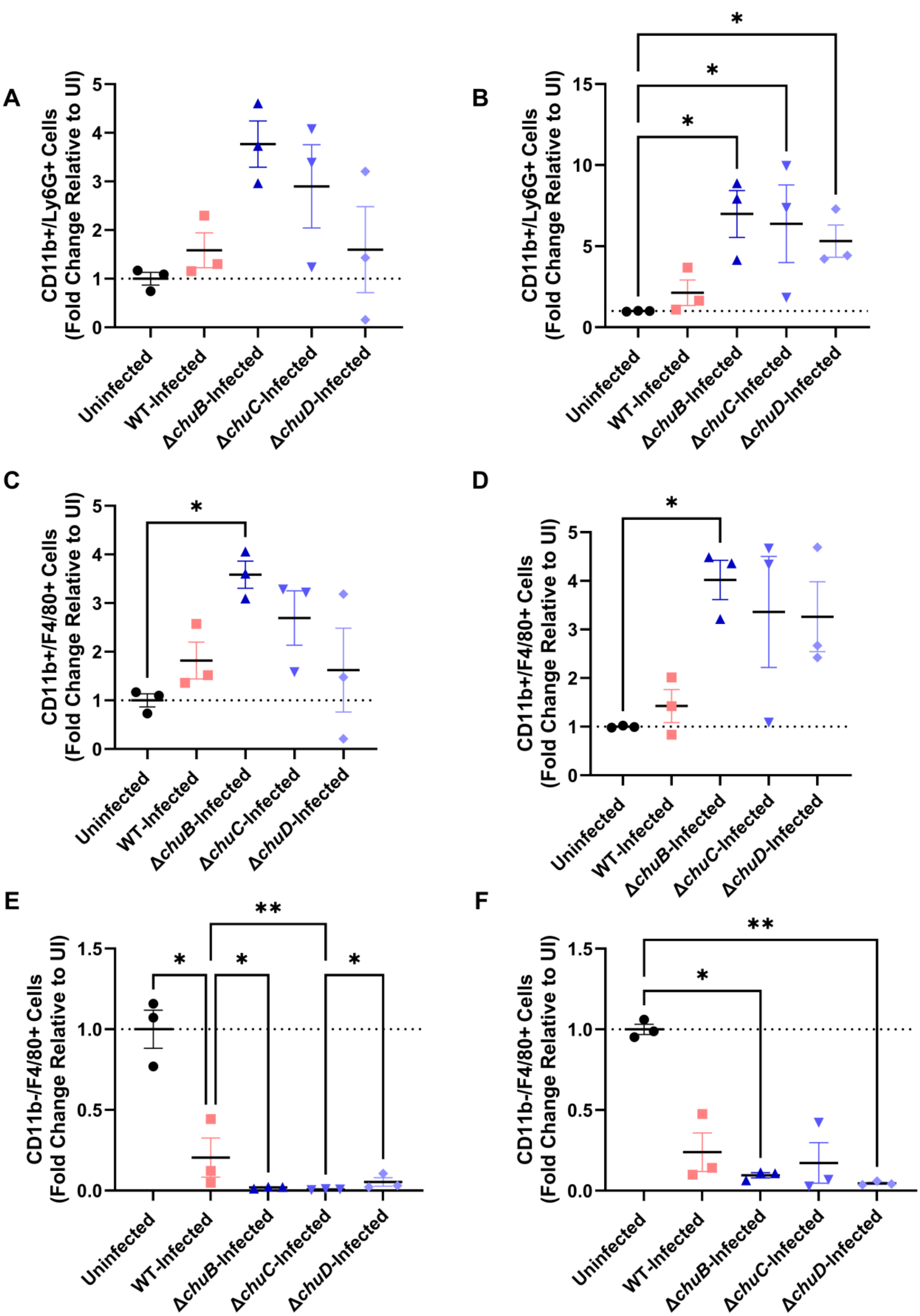

A
CD11b+/Ly6G+ Cells
(Fold Change Relative to UI)
Uninfected
WT-Infected
ΔchuB-Infected
ΔchuC-Infected
ΔchuD-Infected
B
CD11b+/Ly6G+ Cells
(Fold Change Relative to UI)
*
C
CD11b+/F4/80+ Cells
(Fold Change Relative to UI)
*
D
CD11b+/F4/80+ Cells
(Fold Change Relative to UI)
*
E
CD11b-/F4/80+ Cells
(Fold Change Relative to UI)
*
**
F
CD11b-/F4/80+ Cells
(Fold Change Relative to UI)
*
**

Figure S3

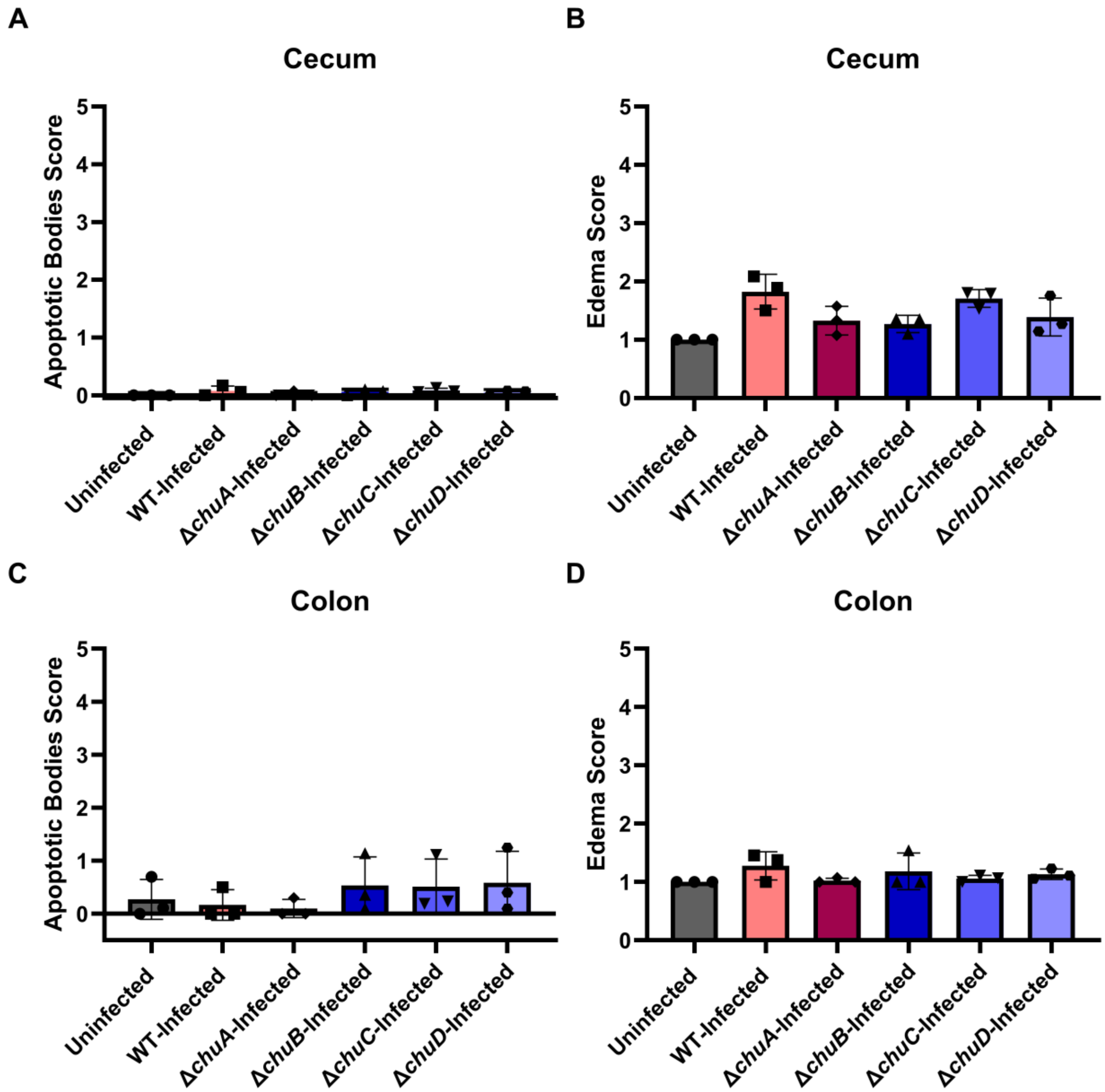

A
Cecum
Apoptotic Bodies Score
0
1
2
3
4
5
Uninfected
WT-Infected
ΔchuA-Infected
ΔchuB-Infected
ΔchuC-Infected
ΔchuD-Infected
B
Cecum
Edema Score
0
1
2
3
4
5
Uninfected
WT-Infected
ΔchuA-Infected
ΔchuB-Infected
ΔchuC-Infected
ΔchuD-Infected
C
Colon
Apoptotic Bodies Score
0
1
2
3
4
5
Uninfected
WT-Infected
ΔchuA-Infected
ΔchuB-Infected
ΔchuC-Infected
ΔchuD-Infected
D
Colon
Edema Score
0
1
2
3
4
5
Uninfected
WT-Infected
ΔchuA-Infected
ΔchuB-Infected
ΔchuC-Infected
ΔchuD-Infected

Figure S4

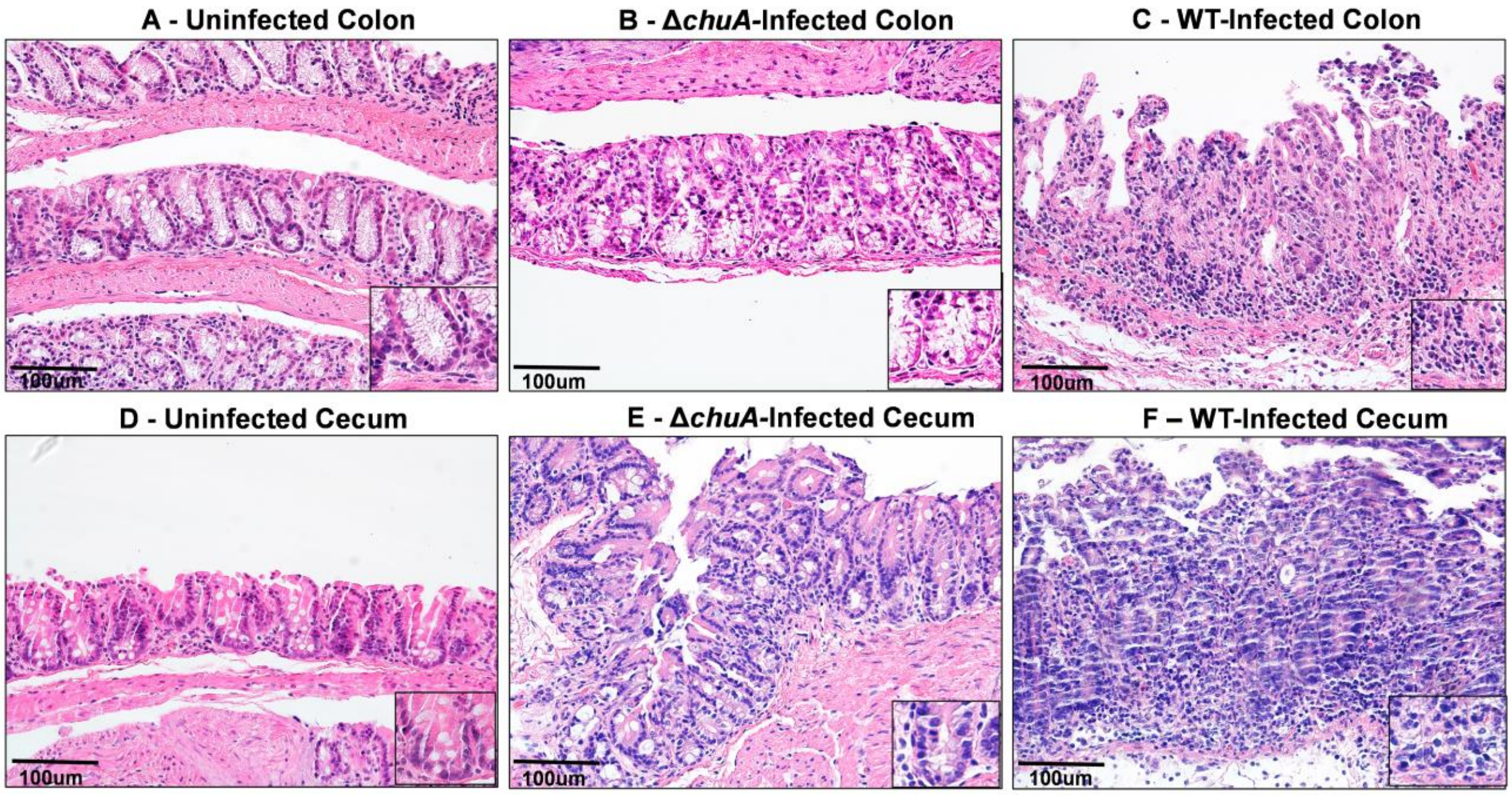
A - Uninfected Colon
B - ΔchuA-Infected Colon
C - WT-Infected Colon
D - Uninfected Cecum
E - ΔchuA-Infected Cecum
F – WT-Infected Cecum
100um
100um
100um
100um
100um
100um

Table S1. Strains used in this study

Strains used in this study	Description	Reference
MAX Efficiency™ DH5α Competent Cells (Catalog No. 18258012)	Cloning strain	Thermo Fisher Scientific
E.coli DH5α pJET1.2Δ*chuA*	*chuA* flanking regions cloned into pJET1.2	This Study
E.coli DH5α pJET1.2Δ*chuB*	*chuB* flanking regions cloned into pJET1.2	This Study
E.coli DH5α pJET1.2Δ*chuC*	*chuC* flanking regions cloned into pJET1.2	This Study
E.coli DH5α pJET1.2Δ*chuD*	*chuD* flanking regions cloned into pJET1.2	This Study
E.coli DH5α pJET1.2Δ*ceuB*	*ceuB* flanking regions cloned into pJET1.2	This Study
E.coli DH5α pJET1.2Δ*ceuD*	*ceuD* flanking regions cloned into pJET1.2	This Study
E.coli DH5α pJET1.2Δ*ceuE*	*ceuE* flanking regions cloned into pJET1.2	This Study
E.coli DH5α pJET1.2Δ*chuA*::*rpsL-cat*	*chuA* flanking regions cloned into pJET1.2 with *H. pylori* *rpsL* and C. coli cat cloned at internal *Bam*HI	This Study
E.coli DH5α pJET1.2Δ*chuB*::*rpsL-cat*	*chuB* flanking regions cloned into pJET1.2 with *H. pylori* *rpsL* and C. coli cat cloned at internal *Bam*HI	This Study
E.coli DH5α pJET1.2Δ*chuC*::*rpsL-cat*	*chuC* flanking regions cloned into pJET1.2 with *H. pylori* *rpsL* and C. coli cat cloned at internal *Bam*HI	This Study
E.coli DH5α pJET1.2Δ*chuD*::*rpsL-cat*	*chuD* flanking regions cloned into pJET1.2 with *H. pylori* *rpsL* and C. coli cat cloned at internal *Bam*HI	This Study
E.coli DH5α pJET1.2Δ*ceuB*::*rpsL-cat*	*ceuB* flanking regions cloned into pJET1.2 with *H. pylori* *rpsL* and C. coli cat cloned at internal *Bam*HI	This Study
E.coli DH5α pJET1.2Δ*ceuD*::*rpsL-cat*	*ceuD* flanking regions cloned into pJET1.2 with *H. pylori* *rpsL* and C. coli cat cloned at internal *Bam*HI	This Study
E.coli DH5α pJET1.2Δ*ceuE*::*rpsL-cat*	*ceuE* flanking regions cloned into pJET1.2 with *H. pylori* *rpsL* and C. coli cat cloned at internal *Bam*HI	This Study
C. jejuni 81-176 SmR (DRH212)	Wild-type strain	[36]
DRH212 Δ*chuA*::*rpsL-cat*	Intermediate deletion-insertion mutant of *chuA*	This Study
DRH212 Δ*chuB*::*rpsL-cat*	Intermediate deletion-insertion mutant of *chuB*	This Study
DRH212 Δ*chuC*::*rpsL-cat*	Intermediate deletion-insertion mutant of *chuC*	This Study
DRH212 Δ*chuD*::*rpsL-cat*	Intermediate deletion-insertion mutant of *chuD*	This Study
DRH212 Δ*ceuB*::*rpsL-cat*	Intermediate deletion-insertion mutant of *ceuB*	This Study
DRH212 Δ*ceuD*::*rpsL-cat*	Intermediate deletion-insertion mutant of *ceuD*	This Study
DRH212 Δ*ceuE*::*rpsL-cat*	Intermediate deletion-insertion mutant of *ceuE*	This Study
DRH212 Δ*chuB* Δ*ceuB*::*rpsL-cat*	In-frame deletion mutant of *chuB* with Intermediate deletion-insertion of *ceuB*	This Study
DRH212 Δ*chuC* Δ*ceuD*::*rpsL-cat*	In-frame deletion mutant of *chuC* with Intermediate deletion-insertion of *ceuD*	This Study
DRH212 Δ*chuD* Δ*ceuE*::*rpsL-cat*	In-frame deletion mutant of *chuD* with Intermediate deletion-insertion of *ceuE*	This Study
DRH212 Δ*chuA*	In-frame deletion mutant of *chuA*	This Study
DRH212 Δ*chuB*	In-frame deletion mutant of *chuB*	This Study
DRH212 Δ*chuC*	In-frame deletion mutant of *chuC*	This Study
DRH212 Δ*chuD*	In-frame deletion mutant of *chuD*	This Study
DRH212 Δ*ceuB*	In-frame deletion mutant of *ceuB*	This Study
DRH212 Δ*ceuD*	In-frame deletion mutant of *ceuD*	This Study

DRH212 Δ*ceuE*	In-frame deletion mutant of *ceuE*	This Study
DRH212 Δ*chuD* Δ*ceuE*	In-frame deletion mutant of *chuD* and *ceuE*	This Study
E. coli DH5α pRY112	*Campylobacter* cloning and expression vector	[37]
E. coli DH5α pRY112*chuA*	*chuA* gene with upstream native promoter region cloned into *Campylobacter* vector pRY112	This Study
E. coli DH5α pRK212.1	Conjugation strain for delivering mobilizable plasmids into *Campylobacter*	[38]
E. coli DH5α pRK212.2 pRY112	Conjugation strain for delivering mobilizable plasmids into *Campylobacter* transformed with pRY112	[39]
E. coli DH5α pRK212.1 pRY112*chuA*	Conjugation strain for delivering mobilizable plasmids into *Campylobacter* transformed with pRY112*chuA*	This Study
DRH212 pRY112	Wild-type strain transformed with empty pRY112	[39]
DRH212 Δ*chuA* pRY112	In-frame deletion mutant of *chuA* transformed with pRY112*chuA*	This Study
DRH212 Δ*chuA* pRY112*chuA*	In-frame deletion mutant of *chuA* transformed with empty pRY112	This Study

Table S1 Citations

36. Hendrixson, D.R., B.J. Akerley, and V.J. DiRita, *Transposon mutagenesis of Campylobacter jejuni identifies a bipartite energy taxis system required for motility*. Mol Microbiol, 2001. **40**(1): p. 214–24.
37. Yao, R., et al., *Construction of new Campylobacter cloning vectors and a new mutational cat cassette*. Gene, 1993. **130**(1): p. 127–30.
38. Figurski, D.H. and D.R. Helinski, *Replication of an origin-containing derivative of plasmid RK2 dependent on a plasmid function provided in trans*. Proc Natl Acad Sci U S A, 1979. **76**(4): p. 1648–52.
39. Davis, L., K. Young, and V. DiRita, *Genetic manipulation of Campylobacter jejuni*. Curr Protoc Microbiol, 2008. **Chapter 8**: p. Unit 8A.2.1–8A.2.17.

Table S2. Primers used in this study

**Primer Name**	**Sequence (5′ → 3′)**
chuA F	CATCAAGCCCAAGCTTTACAA
chuA R	ATCTCCTAGCGCTTAAAGCC
chuA SOE F	TTGCACCCAAATAAAAAAGCTggatccGAATTTAAATACGCATTCTAA
chuA SOE complement	TTAGAATGCGTATTTAAATTCggatccAGCTTTTTTATTTGGGTGCAA
chuA Gibson F	GCGGCCGCTCTAGAACTAGTggatccAGGAAATTCCACTCGTAAAT
chuA Gibson R	GATATCGAATTCCTGCAGCCCGGGggatccGCCCTATAATCACTAAAATT
chuB F	GCCGGCAGATTTCAAATGAG
chuB R	CTTGCAGGATCAAGCACTAC
chuB SOE F	ACGCATTCTAAAATTTTAGTGggatccAGCGCTAGGAGATTTTTATAA
chuB SOE complement	TTATAAAAATCTCCTAGCGCTggatccCACTAAAATTTTAGAATGCGT
chuC F	CATCATAGATGGACGCTTGC
chuC R	GTTTGGTGCCTTGCTGTTTG
chuC SOE F	ATGCTAAAAATACATGATTTAggatccGCTTTAAAGGAGAAAAAAGGA
chuC SOE complement	TCATTTTTTCTCCTTTAAAGCGGAggatccTAAATCATGTATTTTTAGCAT
chuD F	GGATCCATGAAAAAAATTTTAATTATTATGAG
chuD R	CTTCTCGAAAAGGAATTCGC
chuD SOE F	ATGAAAAAAATTTTAATTATTggatccCTAGAAAATAACAATTTTTAA
chuD SOE complement	TTAAAAATTGTTATTTTCTAGggatccAATAATTAAAATTTTTTTCAT
chuB-chuD SOE F	CGCATTCTAAAATTTTAGTGggatccTTTTAATCACCCAATCTTGC
chuD-chuB SOE complement	GCAAGATTGGGTGATTAAAAGGATCCCACTAAAATTTTAGAATGCG
ceuB F	GAAAGCTTTAATCCCCTTGGC
ceuB R	GTGATATTTACCACCAAAAGC
ceuB SOE F	TTGTTTTTTAAGCATATATTGggatccCTTTTGCTAAAAAGGAAAGCT
ceuB SOE complement	AGCTTTCCTTTTTAGCAAAAGggatccCAATATATGCTTAAAAAACAA
ceuD F	AAGTCGTTTTTTACAAATTGC
ceuD R	CTATTTCTTTTAATTTATCAT
ceuD SOE F	ATGATTAAACTAAAAAATATCggatccAAAATTTGTATTTATTTTTAA
ceuD SOE complement	TTAAAAATAAATACAAATTTTggatccGATATTTTTTAGTTTAATCAT
ceuE F	AGTAGTGGAGAAATTTATATC
ceuE R	TCTAAAATTGGAAAATAATCC
ceuE SOE F	ATGAAAAAAATCTTTAGTTTTTggatccATCAAAAACGCTGTAAAATAA
ceuE SOE complement	TTATTTTACAGCGTTTTTGATggatccAAAAACTAAAGATTTTTTTCAT
ceuB Gibson F	TTGTTTTTTAAGCATATATTGggatccAACGACTAAAGTTTTAACAG
ceuB Gibson R	AGCTTTCCTTTTTAGCAAAAGggatccTTATTTATTCAGCAAGTCTT
ceuD Gibson F	ATGATTAAACTAAAAAATATCggatccAACGACTAAAGTTTTAACAG
ceuD Gibson R	TTAAAAATAAATACAAATTTTggatccTTATTTATTCAGCAAGTCTT
ceuE Gibson F	ATGAAAAAAATCTTTAGTTTTTggatccAACGACTAAAGTTTTAACAG
ceuE Gibson R	TTATTTTACAGCGTTTTTGATggatccTTATTTATTCAGCAAGTCTT
