# Supplementary material for: Efficient colonic colonization by Campylobacter jejuni requires the heme receptor ChuA
Source: Infect Immun. 2026 Oct 15;94(11):e00457-26. doi: 10.1128/iai.00457-26 (PMC13750120; doi:10.1128/iai.00457-26)
Supplement: Supplementary file 2 [file iai.00457-26-s0005.docx]

**Figure S1. Complementation of the Δ*chuA* mutant with *chuA* restores heme-dependent iron acquisition and growth.** Wild-type (WT), Δ*chuA* mutant and Δ*chuA* + pRY112*chuA* strains were grown for 48 hours in MH broth containing 320 μM DFOM and with 12.5 μM heme or without heme supplementation. Data are presented as growth relative to growth in MH + 320 μM DFOM and are the result of two independent experiments with triplicate cultures. Statistical analysis was done using unpaired two-tailed Welch's t-test. **p ≤ 0.01

**Figure S2. Innate immune cell populations are recruited in levels similar to wild-type when infected with mutants in ChuB, C, or D.** Murine cecal (A, C, & E) and colonic (B, D, & F) tissues from uninfected, wild-type-(WT) infected, and Δ*chuB-,* Δ*chuC-,* and Δ*chuD-*infected mice were harvested post-infection and were homogenized into single cell suspensions for flow cytometry analysis. Cells were incubated with conjugated antibodies for the immune cell surface markers CD11b, Ly6G, and F4/80. Flow cytometry analysis was performed using FlowJo to identify cell populations positive/negative for CD11b and either Ly6G or F4/80 to differentiate between cell types. The percentage of double positive cells was calculated for each population and fold change relative to the same populations in uninfected mice was reported. Statistical analysis was done using a one-way ANOVA with a Tukey’s multiple comparison test. *p ≤ 0.05, **p ≤ 0.01, ***p ≤ 0.001, ****p ≤ 0.0001

**Figure S3. Infection with the Δ*chuA* mutant does not induce significant apoptosis or edema in the cecum and colon.** Mice were infected with the *C. jejuni* wild-type (WT) strain or one of the mutant strains. Ceca and colons from three mice in each experimental cohort were examined and blind-scored for cecal apoptotic body scores (A), cecal edema scores (B), colon apoptotic body scores (C), and colon edema scores (D). Apoptotic body score was determined by counting apoptotic bodies across all fields and averaging the total per field. Edema was scored across all fields on a scale of 1 to 5, where 1 represents normal tissue, and 5 represents severely inflamed tissue, and then averaged per field. All tissue sections were evaluated under a 20x objective lens. Statistical analysis was done using a one-way ANOVA with a Tukey’s multiple comparison test. * p ≤ 0.05, ** p ≤ 0.01

**Figure S4. Representative H&E-stained sections of the colon and cecum.** Representative hematoxylin and eosin-(HE) stained sections of the colon from (A) uninfected, (B) Δ*chuA* mutant-infected, and (C) wild-type-(WT) infected mice, and of the cecum from (D) uninfected, (E) Δ*chuA*-infected, and (F) WT-infected mice. Images were acquired using a 20X objective. Scale bar, 100 µm.
